# Bidirectional Disulfide Metathesis Enables Recycling of High‐Performance Thermoset Networks

**DOI:** 10.1002/advs.76374

**Published:** 2026-07-13

**Authors:** Bohan Li, Jie Zheng, Daniel Paniroi Situmorang, Jia‐xin Neo, Hongzhi Feng, Jinling Li, Loh Xian‐Jun, Zibiao Li, Teck‐Peng Loh

**Affiliations:** ^1^ Department of Chemistry School of Sciences Great Bay University Dongguan Guangdong China; ^2^ Division of Chemistry and Biological Chemistry, School of Chemistry Chemical Engineering & Biotechnology Nanyang Technological University Singapore Singapore; ^3^ Institute of Materials Research and Engineering (IMRE) Agency for Science, Technology and Research (A*STAR) Singapore Singapore; ^4^ College of Advanced Interdisciplinary Science and Technology Henan University of Technology Zhengzhou Henan China

**Keywords:** bidirectional disulfide metathesis, chemical recycling, polydicyclopentadiene (pDCPD), recyclable thermoset, ring‐opening metathesis polymerization (ROMP)

## Abstract

Polydicyclopentadiene (pDCPD) is a high‐performance thermoset valued for its exceptional mechanical properties, chemical resistance, and thermal stability, enabling broad industrial application. However, its permanent carbon–carbon crosslinked network has long hindered efforts toward sustainable recycling. To overcome this limitation, we present a recycling strategy for pDCPD by incorporating a cyclic disulfide comonomer through ring‐opening metathesis polymerization. The introduction of dynamic disulfide bonds enables thermal reprocessing via associative bond exchange and chemical depolymerization into disulfide‐terminated oligomers that can be repolymerized without loss of performance. In addition to enabling recyclability, the disulfide monomer containing an aromatic ring enhances mechanical performance of pDCPD network in toughness and stiffness. This work provides a route to dual‐mode recyclable, high‐performance hydrocarbon thermosets and establishes bidirectional disulfide metathesis as a practical tool for thermoset circularity.

## Graphical abstract

A cyclic aromatic disulfide comonomer enables dual‐mode recyclable pDCPD thermosets through bidirectional disulfide metathesis. The resulting networks combine enhanced mechanical performance with thermal reprocessability and chemical depolymerization into reusable oligomeric fragments, allowing regeneration of high‐performance thermosets with minimal loss of structural and mechanical integrity.

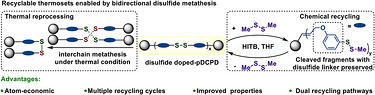

## 1 Introduction

Thermosets represent a fundamental class of polymeric materials, widely utilized in structural and functional applications due to their exceptional mechanical strength, thermal stability, and chemical resistance [1, 2, 3, 4, 5, 6]. However, their permanently crosslinked networks pose significant barriers to end‐of‐life recovery, limiting compatibility with circular materials strategies [7, 8, 9, 10, 11, 12]. Among high‐performance thermosets, pDCPD is particularly notable for its outstanding toughness and chemical inertness, enabled by its polyolefin‐like backbone and high crosslink density [13, 14, 15, 16, 17, 18]. These same features, however, render conventional recycling approaches—such as pyrolysis—energetically intensive and environmentally unsustainable [19]. Overcoming the intrinsic irreversibility of pDCPD's network remains a central challenge in advancing recyclable thermoset technologies [20].

**FIGURE 1:**
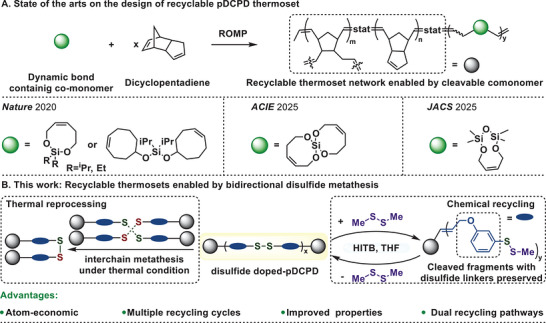
Background and design concept. (A). State‐of‐the‐art recycling of polydicyclopentadiene (pDCPD) is enabled by incorporating cleavable comonomers within the polymer network (B). A disulfide‐functionalized monomer copolymerizes efficiently with dicyclopentadiene via ring‐opening metathesis polymerization (ROMP), embedding dynamic covalent disulfide bonds into the pDCPD network. The resulting thermoset demonstrates dual recyclability by thermal reprocessing and chemical recycling.

Covalent adaptable networks (CANs), including the vitrimer subclass, offer a compelling strategy for imparting reprocessability to permanently crosslinked polymers [21, 22, 23, 24, 25, 26]. These systems leverage dynamic covalent chemistries that mediate bond exchange reactions without disrupting overall network connectivity. In particular, associative exchange mechanisms—such as transesterification [27, 28], transamination [29], transthioetherification [30], imine exchange [31], silyl ether exchange [32, 33, 34] and disulfide exchange [35, 36, 37, 38, 39]—enable thermosets to be reprocessed in the solid state while preserving crosslink density and mechanical integrity. In parallel, complementary approaches have shown that the incorporation of cleavable bonds into thermoset backbones allows for chemically triggered depolymerization under mild conditions, expanding the design space for degradable and recyclable networks [25, 26, 29, 30]. Johnson and colleagues pioneered this approach in pDCPD by introducing cleavable comonomers that enabled selective degradation [40, 41, 42] (Figure 1A). These advances collectively underscore the potential of molecular design to reconcile reprocessability and degradability with the demanding performance requirements of thermoset materials. Nonetheless, further progress is needed to broaden the chemical scope of cleavable motifs and to develop efficient, reproducible recycling strategies.

Building on our previous development of a bidirectional disulfide metathesis platform [43], we sought to extend dynamic disulfide chemistry to pDCPD thermosets to enable recyclability. We designed a cyclic disulfide monomer capable of copolymerizing with dicyclopentadiene (DCPD) via ring‐opening metathesis polymerization (ROMP), yielding a disulfide‐doped pDCPD network. We systematically investigated the synthesis, degradation, and regeneration of these materials (Figure 1B). Notably, the incorporation of a phenyl‐substituted disulfide unit introduced *π–π* stacking interactions and rigidity that enhanced the mechanical performance of the network, achieving up to a 90% increase in tensile strength relative to unmodified pDCPD. More critically, the dynamic nature of the disulfide bonds enabled dual‐mode recyclability: thermal reprocessing through bond exchange and chemical depolymerization into functional oligomers. Mechanical testing of the regenerated materials revealed full retention of mechanical properties, confirming that dynamic bond design can enable reversible thermoset architectures without compromising structural integrity. These findings provide a robust framework for designing reprocessable and chemically recyclable thermosets and highlight disulfide metathesis as a promising tool for advancing sustainable polymer network architecture.

## 2 Results and Discussion

**FIGURE 2:**
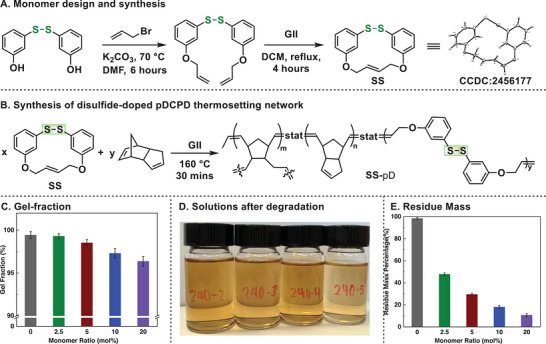
Monomer design, polymer synthesis, and degradation tests. (A). Synthesis of the cyclic disulfide monomer (**SS**) bearing a vinyl group for subsequent copolymerization with dicyclopentadiene (B). Copolymers were prepared by ring‐opening metathesis polymerization (ROMP) of DCPD with varying feed ratios of the cyclic disulfide monomer (**SS**) (C). Gel fractions of disulfide‐modified pDCPD networks after 48 h of immersion in tetrahydrofuran (THF), demonstrating the formation and stability of the crosslinked thermoset network. (D) **SS**‐doped pDCPD networks exhibited degradation in tetrahydrofuran (THF) at 70°C in the presence of the radical initiator hydroxy(tosyloxy)iodobenzene (HTIB) and dimethyl disulfide (DMDS) (E). Quantification of residual mass following treatment with hydroxy(tosyloxy)iodobenzene (HTIB) and dimethyl disulfide (DMDS) in THF at 70°C for 12 h, illustrating a clear dependence of network dissolution on the molar fraction of the incorporated **SS** comonomer.

To initiate our study, we designed and synthesized a cyclic disulfide monomer through a straightforward two‐step synthetic route in 61%, which was subsequently copolymerized with dicyclopentadiene at different molar ratios (2.5, 5, 10, 20 mol%) via ring‐opening metathesis polymerization (ROMP) to yield a series of degradable thermosets (Figure 2B). The obtained polymers were then subjected to swelling experiments, and the gel fractions for all the samples exceeded 95%, indicating the formation of the crosslinked thermoset network (Figure 2C). Building on our group's previously established model of bidirectional disulfide metathesis, we hypothesized that disulfide linkages embedded within the thermoset backbone could undergo exchange reactions with a small‐molecule disulfide, leading to network cleavage and formation of soluble fragments.

Consistent with this hypothesis, exposure of the disulfide‐doped pDCPD to a cost‐effective hypervalent iodine initiator, hydroxy(tosyloxy)iodobenzene (HTIB), and dimethyl disulfide (DMDS) in THF at 70°C resulted in visible degradation of the thermosets after 6 h of stirring (Figure 2D). Residual mass quantification revealed that higher incorporation ratios of the disulfide comonomer led to more extensive degradation (Figure 2E).

We subsequently carried out a comprehensive evaluation of the functional performance of disulfide‐doped poly(dicyclopentadiene) in comparison to the unmodified thermoset. This assessment focused on both thermal and mechanical properties, aiming to elucidate the impact of disulfide monomer incorporation on the resulting polymer network. For clarity, samples were designated as **SS**‐pD‐xx, where “xx” denotes the molar percentage of the disulfide‐containing monomer incorporated into the formulation.

**FIGURE 3:**
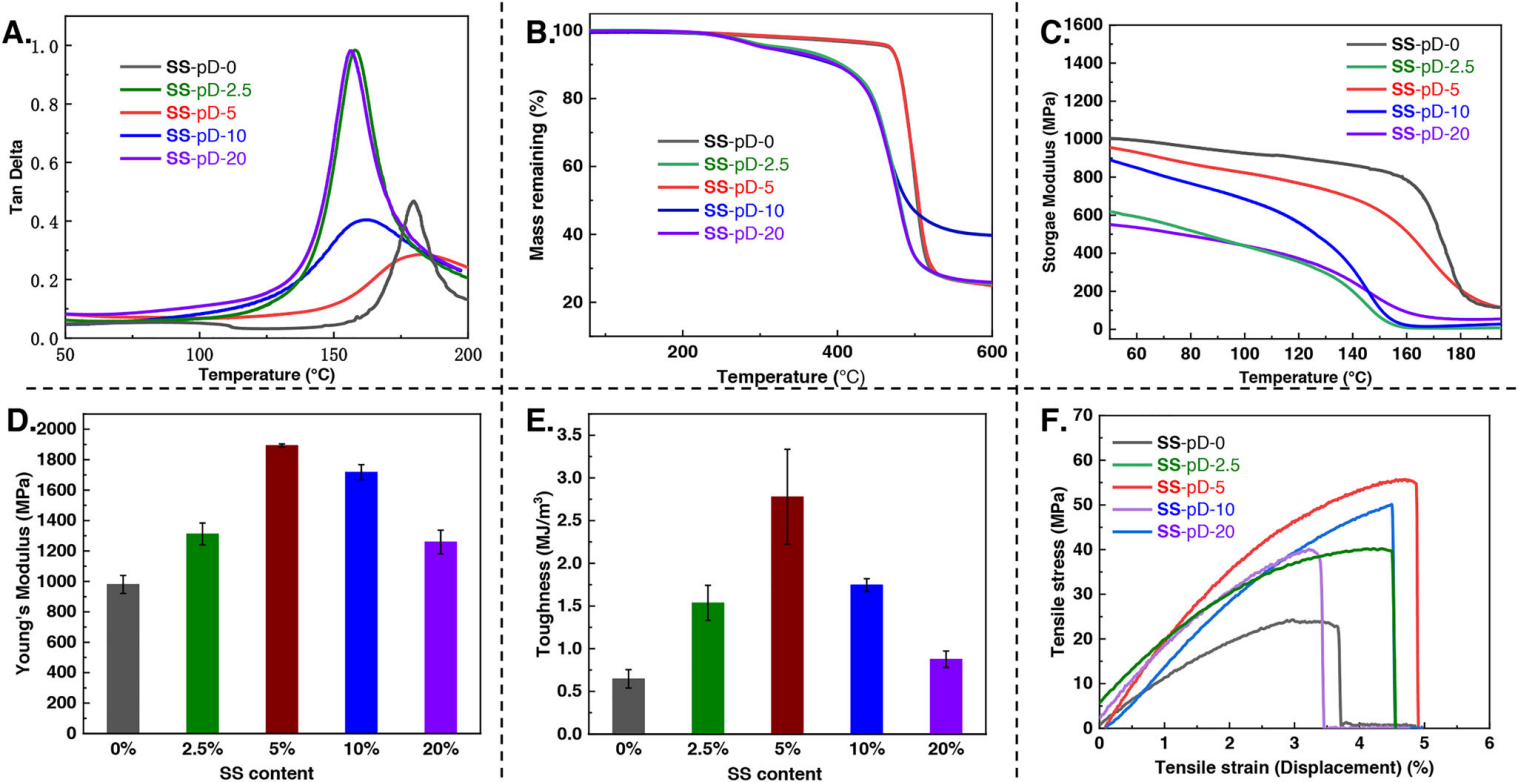
Functional evaluation of disulfide‐doped pDCPD. (A) The glass transition temperature (Tg) exhibits a non‐linear dependence on **SS** content, reaching a maximum at 5 mol%, as evidenced by dynamic mechanical analysis (DMA). (B) Thermogravimetric analysis (TGA) further corroborates this trend, revealing a similar non‐monotonic relationship, with thermal stability reaching a maximum at a 5 mol% **SS** doping level. (C) All **SS**‐doped networks exhibited decreased storage modulus relative to pristine pDCPD, yet retained characteristic thermoset behavior with non‐zero modulus at elevated temperatures; (D) Young's modulus increased with **SS** incorporation, reaching a maximum at 5 mol%, consistent with the Tg trend observed in DMA; (E) Toughness also improved at moderate **SS** loadings, with the highest value observed at 5 mol%; (F) Representative tensile stress–strain curves showing similar strain at break across all samples, with increased strength and stiffness at intermediate **SS** content.

Interestingly, DMA analysis revealed a non‐monotonic trend in the glass transition temperature (Tg) as a function of **SS** content. Specifically, Tg increased from 158.3°C at 2.5 mol% **SS** to a maximum of 184.3°C at 5 mol%, then decreased to 156.4°C at 20 mol% (Figure 3A). The non‐monotonic dependence of the thermomechanical and mechanical properties on **SS** content can be rationalized by two competing structural effects. As **SS** content increases, more aromatic disulfide units are incorporated into the pDCPD network. The rigid phenyl rings can restrict local segmental motion and increase chain stiffness, while WAXS analysis (Figure S9) shows enhanced broad scattering in the 22°–26° region, corresponding to d‐spacings of approximately 4.0–3.4 Å. This result is consistent with increased short‐range aromatic packing or possible *π–π* interactions in the **SS**‐containing networks. These effects contribute to the increased Tg, Young's modulus, and toughness observed at moderate **SS** loading, especially for **SS**‐pD‐5. However, excessive **SS** incorporation also introduces a higher fraction of flexible disulfide‐containing segments and cleavable/dynamic sites, which reduces the effective compactness of the pDCPD network. Apparent crosslinking‐density calculations (Table S8) based on the rubbery storage modulus show that **SS**‐pD‐20 has a lower effective crosslinking density than **SS**‐pD‐5. Therefore, at higher **SS** content, the decrease in effective crosslinking density and the increase in segmental mobility outweigh the reinforcing effects of aromatic rigidity and short‐range packing, resulting in reduced Tg and modulus. Thus, the maximum performance at 5 mol% **SS** is attributed to an optimal balance between aromatic‐unit‐induced reinforcement, possible short‐range aromatic packing, reversible disulfide‐mediated energy dissipation, and preservation of sufficient effective crosslinking density. This non‐monotonic trend was corroborated by TGA analysis, which showed a similar variation in thermal stability (Figure 3B), with the onset of degradation temperature ranging from 307°C to 466°C and reaching a maximum at 5 mol% **SS** incorporation. These findings highlight the dual role of disulfide crosslinks in tuning thermomechanical properties—initially reinforcing the network but ultimately diminishing stability at excessive loading levels.

A similar trend to that observed in the thermal analysis was reflected in the mechanical performance of the materials. Tensile testing showed a marked increase in Young's modulus at 5 mol% **SS** incorporation (Figure 3D), indicating enhanced stiffness and reduced chain mobility at this intermediate doping level. However, further increasing the **SS** content led to a decline in modulus, consistent with the Tg trend. Furthermore, toughness was significantly improved across all **SS**‐doped samples (Figure 3E), emphasizing the role of dynamic disulfide bonds in dissipating mechanical energy and enhancing resistance to crack initiation and propagation through reversible bond exchange mechanisms.

**FIGURE 4:**
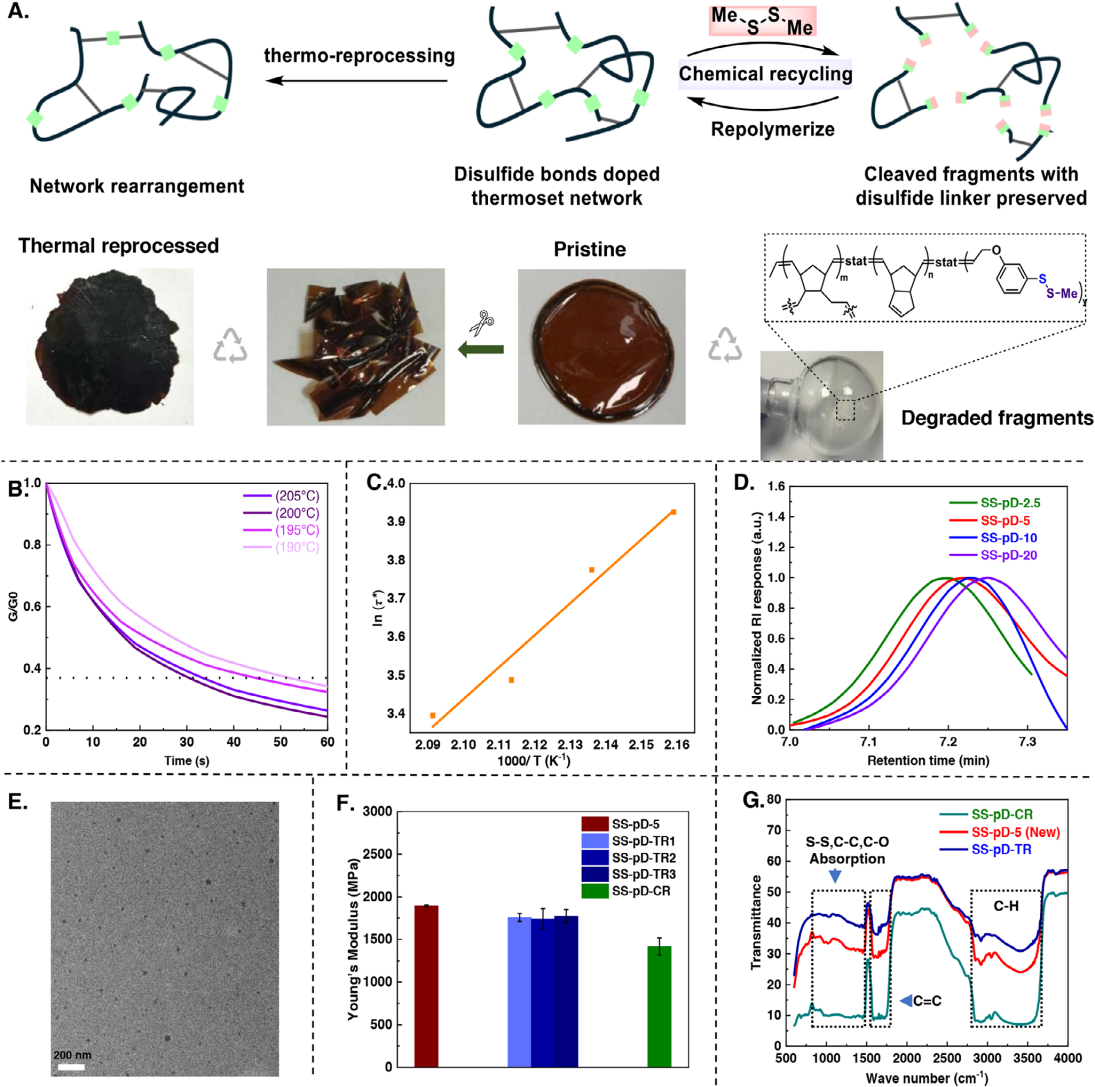
Recycling Experiments. (A) Schematic illustration of disulfide‐doped pDCPD via two pathways: thermo‐reprocessing through dynamic bond exchange and chemical degradation into disulfide‐containing fragments followed by repolymerization; photographic demonstration of thermal recycling of 5 mol% **SS**‐doped pDCPD, repolymerized material recovered from chemically degraded fragments and chemically degraded fragments recovered from solution via recrystallization, (B) Stress‐relaxing curves show exponential decrease over time of relaxation modulus of 5 mol% **SS**‐doped pDCPD; (C) Fitting of the relaxation time to Arrhenius’ equation yields an activation energy of 69 kJ/mol; (D) GPC traces of degradation products reveal a shift to higher retention times with increasing **SS** content, indicating formation of smaller oligomeric species; (E) TEM image of chemically cleaved fragments from 5 mol% **SS**‐doped pDCPD, showing an average particle size of ∼20 nm; (F) Comparison of Young's modulus for pristine, thermally reprocessed, and chemically recycled 5 mol% **SS**‐doped pDCPD; (G) IR spectra of pristine, thermally reprocessed and chemically recycled samples indicate preservation of chemical structure post‐recycling.

To further explore the recycling pathways, we selected the 5 mol% disulfide‐incorporated formulation (**SS**‐pD‐5)—previously shown to exhibit excellent thermal and mechanical properties—as the representative system for recyclability investigations (Figure 4A). To validate the dynamic covalent behavior of the disulfide‐functionalized pDCPD network, Stress‐relaxation experiments were performed at elevated temperatures (190°C, 195°C, 200°C, and 205°C) using dynamic mechanical analysis (DMA) to evaluate the material's ability to dissipate internal stress. As illustrated in Figure 4B, the relaxation modulus decreased exponentially over time, characteristic of vitrimeric behavior enabled by thermally activated disulfide exchange reactions within the cross‐linked network. The relaxation time (τ^*^), defined by the Maxwell model as the time required for stress to decay to 1/e (36.7%) of its initial value, was extracted at each temperature.

As anticipated, bond exchange dynamics accelerated with increasing temperature, yielding τ^*^ values ranging from 51 s at 190°C to 29 s at 205°C. Fitting the temperature‐dependent relaxation times to the Arrhenius equation yielded an activation energy (E_a_) of 69 kJ/mol for the disulfide exchange process (Figure 4C and Figure S10). This value is slightly lower than those reported for comparable networks [27, 44, 45, 46, 47], which may be attributed to the inherent flexibility and favorable kinetics of the disulfide exchange mechanism. Capitalizing on the reactivity of the dynamic disulfide bonds, we further assessed the material's reprocessability. Following mechanical failure, the samples were cut into small fragments and thermally reprocessed into uniform films via hot pressing at 180°C under 20 bar for 20 min. This reprocessing procedure was repeated for three consecutive cycles, with the resulting samples denoted as **SS**‐pD‐TR1, **SS**‐pD‐TR2, and **SS**‐pD‐TR3, respectively.

Having established the excellent thermal reprocessability of the disulfide‐doped pDCPD network, we next explored its chemical degradability and potential for chemical recycling. THF solutions obtained from the degradation experiments (Figure 2D) were concentrated by solvent evaporation, and the resulting residues were subjected to low‐temperature precipitation/filtration, which effectively removed the characteristic brown coloration from Grubbs’ catalyst. This process afforded soluble oligomeric fragments in 74 wt.% recovery, providing reusable polymer‐derived fragments for subsequent reconstitution with fresh DCPD. The fragments were characterized by an orthogonal set of techniques. Gel permeation chromatography (GPC) analysis of the soluble fractions revealed a clear inverse correlation between **SS** content and molecular weight of the degradation products, with weight‐average molar masses ranging from approximately 1–6 kDa (Figure 4D and Table S7). Transmission electron microscopy (TEM) of the degradation mixture derived from the **SS**‐pD‐5 sample further confirmed the formation of well‐dispersed nanoscale fragments with an average particle diameter of ∼20 nm (Figure 4E).

To further characterize the chemically degraded products, ^1^H NMR, DOSY NMR, and DLS analyses were performed. The ^1^H NMR spectrum showed characteristic aliphatic and olefinic signals from pDCPD‐derived fragments together with aromatic signals associated with the **SS** comonomer (Figure S13). DOSY NMR showed comparable diffusion behavior for the major pDCPD‐ and **SS**‐derived resonances on the order of 10^−9^ cm^2^ s^−^^1^, supporting their assignment to the same soluble oligomeric/polymeric fragment population rather than independent small‐molecule residues (Figure S14). Because the **SS** comonomer was incorporated at relatively low loading, chemical degradation is expected to generate large soluble oligomeric fragments rather than small molecules. Consistently, DLS revealed nanoscale species with an average hydrodynamic diameter of approximately 20 nm (Figure S15). These findings validate the effectiveness of the chemical depolymerization strategy and highlight the tunability of fragment size through **SS** incorporation, offering a promising route for controlled degradation and chemical recycling of crosslinked thermoset networks.

Notably, the degradation products retained chemically reactive features, including pendant cyclopentene groups, exchangeable alkene‐containing crosslinks, and terminal disulfide functionalities resulting from metathesis‐driven cleavage. This prompted the hypothesis that such oligomeric fragments could be repolymerized to regenerate functional disulfide‐doped pDCPD materials. To test this, the degradation mixture was blended with fresh DCPD in dichloromethane (DCM), followed by ring‐opening metathesis polymerization under identical conditions to the original network. This process successfully yielded a regenerated thermoset film (Figure S16). Gas chromatography–mass spectrometry (GC‐MS) of the film surface confirmed the presence of residual dimethyl disulfide, consistent with the proposed reaction pathway (Figure S17). As previously modeled, the volatilization of dimethyl disulfide under heating and vacuum conditions shifts the equilibrium of disulfide exchange reactions toward the formation of symmetrical disulfides, thereby promoting network reconstitution.

More importantly, tensile testing of materials recovered from both recycling pathways demonstrated near‐complete retention of mechanical performance (Figure 4F and Figures S12 and S18). The reprocessed samples exhibited Young's modulus and stress–strain behavior closely matching those of the original thermoset. Additionally, FTIR spectra of pristine **SS**‐pD‐5, thermally reprocessed **SS**‐pD‐TR, and chemically reconstituted **SS**‐pD‐CR samples showed similar characteristic absorption bands, including C─H stretching, residual C═C vibration, and the overlapped S─S/C─C/C─O absorption region. The absence of obvious new absorption bands or loss of characteristic peaks suggests that the major chemical features of the **SS**‐doped pDCPD network are largely preserved after thermal reprocessing and chemical reconstitution. (Figure 4G). These results confirm that disulfide‐doped pDCPD can be effectively recycled and reconstituted into high‐performance thermosets with minimal loss of mechanical and chemical integrity.

## 3 Conclusion

We have established a dual‐mode recycling strategy for high‐performance pDCPD thermosets based on the incorporation of a cyclic aromatic disulfide comonomer. ROMP of DCPD with the SS comonomer yields elastically active crosslinked networks, and the aromatic disulfide unit reinforces the network through a combination of chain rigidity, short‐range aromatic packing, dynamic‐bond energy dissipation, and preserved crosslink density. At the optimum 5 mol% loading, the material reaches *T*_g_ = 184.3°C and Young's modulus = 1732 MPa, surpassing pristine pDCPD as well as previously reported cleavable‐comonomer pDCPD systems.

The same disulfide handle enables two complementary recycling pathways within the same material. Solid‐state thermal reprocessing proceeds through associative disulfide exchange (E_a_ = 69 kJ mol^−^^1^) and supports at least three reprocessing cycles with retained mechanical performance. Chemical fragmentation/reconstitution proceeds by bidirectional disulfide metathesis with a small‐molecule DMDS shuttle, giving soluble oligomeric fragments (∼20 nm hydrodynamic diameter; characterized by GPC, TEM, DLS, ^1^H NMR, and DOSY NMR) that can be recombined with fresh DCPD by ROMP to give a regenerated dynamic thermoset (**SS**‐pD‐CR) with mechanical performance close to that of the parent material.

More broadly, this work extends dynamic disulfide chemistry to a robust hydrocarbon thermoset that has previously been inaccessible to vitrimer‐style reprocessing and demonstrates that bidirectional disulfide metathesis is a practical tool for combining thermal reprocessing with chemical fragment recovery in a single high‐performance pDCPD network. We anticipate that the cyclic disulfide comonomer concept can be transferred to other ROMP‐active thermoset platforms, and that the combination of an aromatic disulfide unit and a small‐molecule disulfide shuttle will provide a general route to dual‐mode recyclable, high‐performance crosslinked polymers.

## Author Contributions

**Daniel Paniroi Situmorang**: methodology, validation. **Jie Zheng**: investigation, data curation, writing – review and editing, writing – original draft. **Zibiao Li**: writing – original draft, writing – review and editing, conceptualization, resources, supervision, funding acquisition, project administration. **Bohan Li**: conceptualization, investigation, methodology, writing – original draft, writing – review and editing, data curation, software, visualization, validation. **Jinling Li**: resources, validation. **Hongzhi Feng**: formal analysis, data curation, investigation, methodology. **Teck‐Peng Loh**: funding acquisition, writing – original draft, conceptualization, writing – review and editing, resources, supervision, project administration. **Jia‐xin Neo**: methodology, validation. **Loh Xian‐jun**: writing – review and editing, validation.

## Conflicts of Interest

The authors declare no conflicts of interest.

## Supporting information




**Supporting File**: advs76374‐sup‐0001‐SuppMat.docx.

## Data Availability

The data that support the findings of this study are available in the supplementary material of this article.
