# Supplementary material for: Bidirectional Disulfide Metathesis Enables Recycling of High‐Performance Thermoset Networks
Source: Adv Sci (Weinh). 2026 Jul 13:e76374. Online ahead of print. doi: 10.1002/advs.76374 (PMC13360114; doi:10.1002/advs.76374)
Supplement: Supplementary file 1 — Supporting File: advs76374‐sup‐0001‐SuppMat.docx. [file ADVS-9999-e76374-s001.docx]

Supplementary Materials for

**Bidirectional Disulfide Metathesis Enables Recycling of High-Performance Thermoset Networks**

Bohan Li^2^, Jie Zheng^3^, Daniel Paniroi Situmorang^2^, Jia-xin Neo^2^, Hongzhi Feng^3^, Jinling Li^4^, Loh Xian Jun^3^, Zibiao Li^3^*, Teck-Peng Loh^1,2,4^*

^1^Department of Chemistry, School of Sciences, Great Bay University, Dongguan, Guangdong, 523000, China.

^2^Division of Chemistry and Biological Chemistry, School of Chemistry Chemical Engineering & Biotechnology, Nanyang Technological University, 21 Nanyang Link, 637371, Singapore.

^3^Institute of Materials Research and Engineering, Agency for Science, Technology, and Research (A*STAR), Singapore 138634, Republic of Singapore.

^4^100 Lianhua Street, Zhongyuan District, Henan University of Technology, Zhengzhou, 450001, China.

*Corresponding authors. Email: teckpeng@ntu.edu.sg (T.P.L.); lizb@imre.astar.edu.sg (Z. L.)

**The PDF file includes:**

Figure. S1 to S18

Tables S1 to S10

References

**Table of contents**

I. General Methods

II. Synthesis and characterization of the monomer

III. Synthesis of the polymers

IV. Swelling experiments and gel content tests

V. Degradation tests and quantification of residue mass

VI. Functional performance characterization of polymers

VII. Wide-Angle X-ray Scattering (WAXS) Characterization

VIII. Malleability and reprocessability of SS-doped pDCPD samples

IX. Recovery of degraded SS-doped pDCPD fragments and characterization

X. Recycling of pDCPD fragments and characterization

XI. Comparison with State-of-the-Art pDCPD Recycling Systems

XII. References

**I. General Methods**

**NMR Spectroscopy**

Proton (¹H) and carbon (¹³C) nuclear magnetic resonance (NMR) spectra were recorded using Bruker 400 MHz spectrometers. Spectra were acquired in the indicated deuterated solvents, with ¹H chemical shifts referenced to residual solvent peaks (e.g., CDCl₃ at δ 7.26 ppm) and ¹³C chemical shifts referenced to solvent resonance (e.g., CDCl₃ at δ 77.16 ppm). Multiplicities are denoted as s (singlet), d (doublet), t (triplet), q (quartet), and m (multiplet). The number of protons contributing to each signal is indicated by “nH”, and coupling constants are reported as J values in hertz.

**FTIR Spectroscopy**

Fourier-transform infrared (FTIR) spectra were collected using a Nicolet iS50 FTIR spectrometer in attenuated total reflection (ATR) mode. Scans were recorded over a wavenumber range of 4000–400 cm⁻¹ at room temperature.

**Thermogravimetric Analysis (TGA)**: Thermal stability was evaluated using a TA Instruments Q500 system. Approximately 10 mg of sample was heated from 30 to 800 °C at a rate of 20 °C/min under a nitrogen flow (60 mL/min). The onset of decomposition was defined as the temperature corresponding to 5% weight loss (T₅%).

**Dynamic Mechanical Analysis (DMA)**

Glass transition temperatures (Tg) , viscoelastic behavior and thermomechanical properties were measured using a TA Instruments Q800 in single cantilever mode. Rectangular specimens (20 mm × 4 mm × 0.15 mm) were tested from 30 to 150 °C at a frequency of 1 Hz and a temperature ramp rate of 3 °C/min. A 20 μm oscillation amplitude was applied. Storage modulus (E′) and tan δ were recorded as a function of temperature.

**Stress Relaxation:** To examine stress relaxation behavior, samples were preloaded with 1 × 10⁻³ N to eliminate slack and equilibrated for 3 minutes after reaching the target temperature (160–190 °C). A constant strain of 5% was then applied, and the decay of the relaxation modulus was monitored over time.

**Mechanical Testing**

Tensile properties were evaluated using an Instron 5567 universal testing machine at room temperature. Specimens (30 mm × 5 mm × 0.15 mm) were prepared by injection molding and tested with a 15 mm gauge length and a crosshead speed of 5 mm/min. Reported values include Young’s modulus, tensile strength, and elongation at break, with results averaged over three replicates and presented with standard deviations.

**GC-MS Analysis**

Gas chromatography–mass spectrometry (GC-MS) was carried out using an Agilent 7890A system under a nitrogen atmosphere with a flow rate of 1 mL/min. The temperature program began with a 1-minute hold at 50 °C, followed by heating at 5 °C/min to 300 °C, where it was maintained for 5 minutes.

**Materials and Reagents**

All reagents and solvents were obtained commercially and used without further purification unless otherwise noted. Flash column chromatography was performed on silica gel (Merck 40–63 µm, 60 Å). High-resolution mass spectra (HRMS) were obtained on a Waters G2-XS QTof mass spectrometer using electrospray ionization (ESI) unless stated otherwise.

**II. Synthesis and characterization of the monomer**

**Figure S1. Monomer synthesis step 1**

*Procedure for Figure S1:* To a 100 mL oven-dried round-bottom flask equipped with a magnetic stir bar, 3,3'-disulfanediyldiphenol (**1a**, 2.5 g, 10 mmol, 1.0 equiv) was dissolved in N,N-dimethylformamide (DMF, 30 mL). Potassium carbonate (3.31 g, 24 mmol, 2.4 equiv) was added in one portion, followed by stirring at 70 °C for 30 minutes. Subsequently, allyl bromide (2.90 g, 24 mmol, 2.4 equiv) was added dropwise, and the reaction mixture was stirred at 70 °C for an additional 5.5 hours. Upon completion, the reaction was quenched with water and extracted with ethyl acetate (3 × 25 mL). The combined organic layers were washed with brine, dried over anhydrous sodium sulfate, filtered, and concentrated under reduced pressure. The crude product was purified by column chromatography to afford the desired product **1b**.

1,2-bis(3-(allyloxy)phenyl)disulfane (**1b**)

The product was isolated by column chromatography (hexane/ethyl acetate = 20:1, Rf = 0.6) as a yellow oil in 91% yield.

^1^H NMR (400 MHz, CDCl_3_) δ 7.26 – 7.20 (m, 1H), 7.12 (q, *J* = 5.3, 3.6 Hz, 2H), 6.86 – 6.77 (m, 1H), 6.15 – 5.95 (m, 1H), 5.47 – 5.35 (m, 1H), 5.30 (d, *J* = 10.5 Hz, 1H), 4.53 (d, *J* = 6.0 Hz, 2H).

^13^C NMR (101 MHz, CDCl_3_) δ 159.12, 138.27, 132.95, 129.92, 119.74, 117.95, 113.96, 113.34, 68.90.

HRMS (ESI): Calcd for C_18_H_19_O_2_S_2_ [M+H]^+^: 331.0826; found: 331.0827.


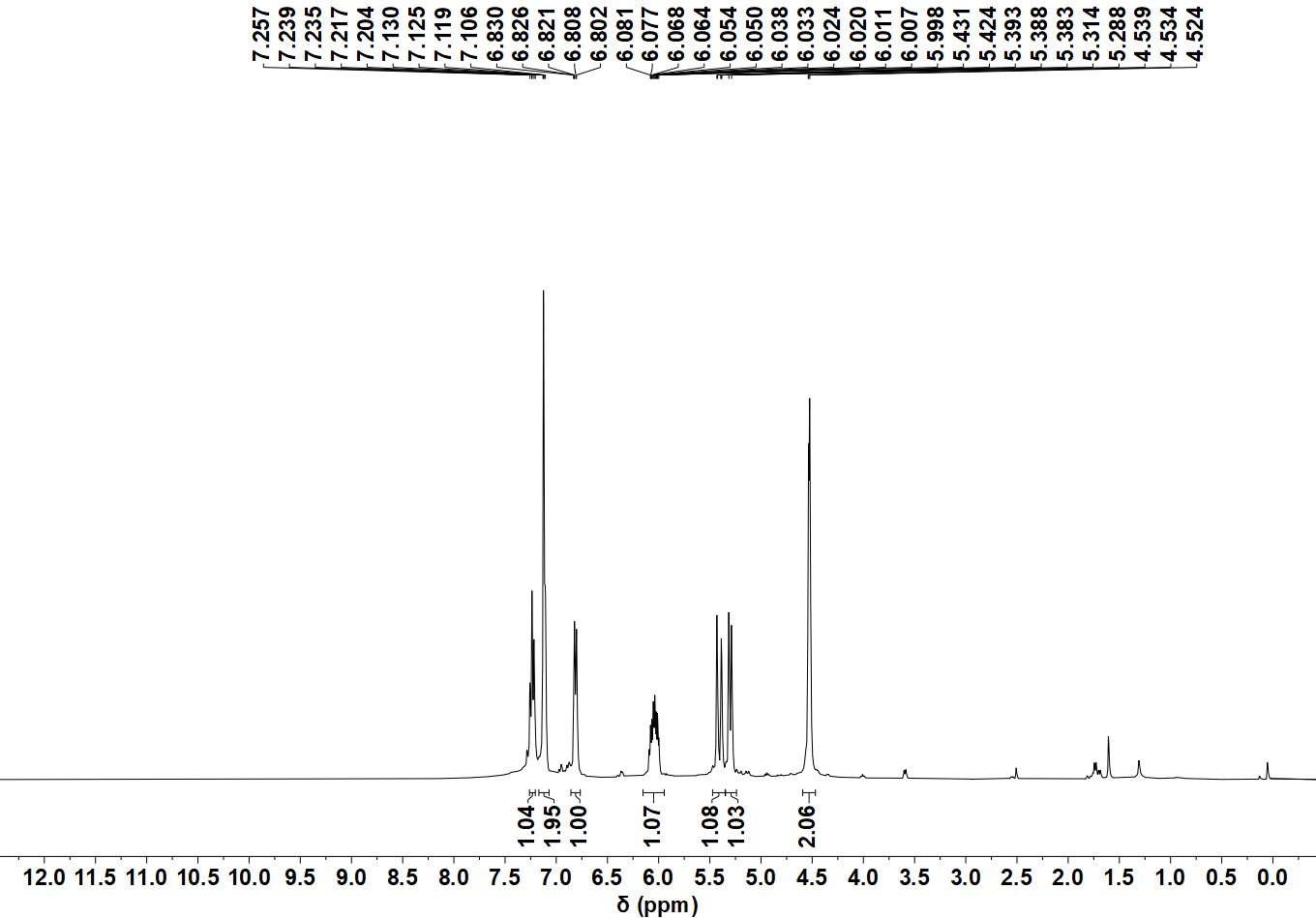


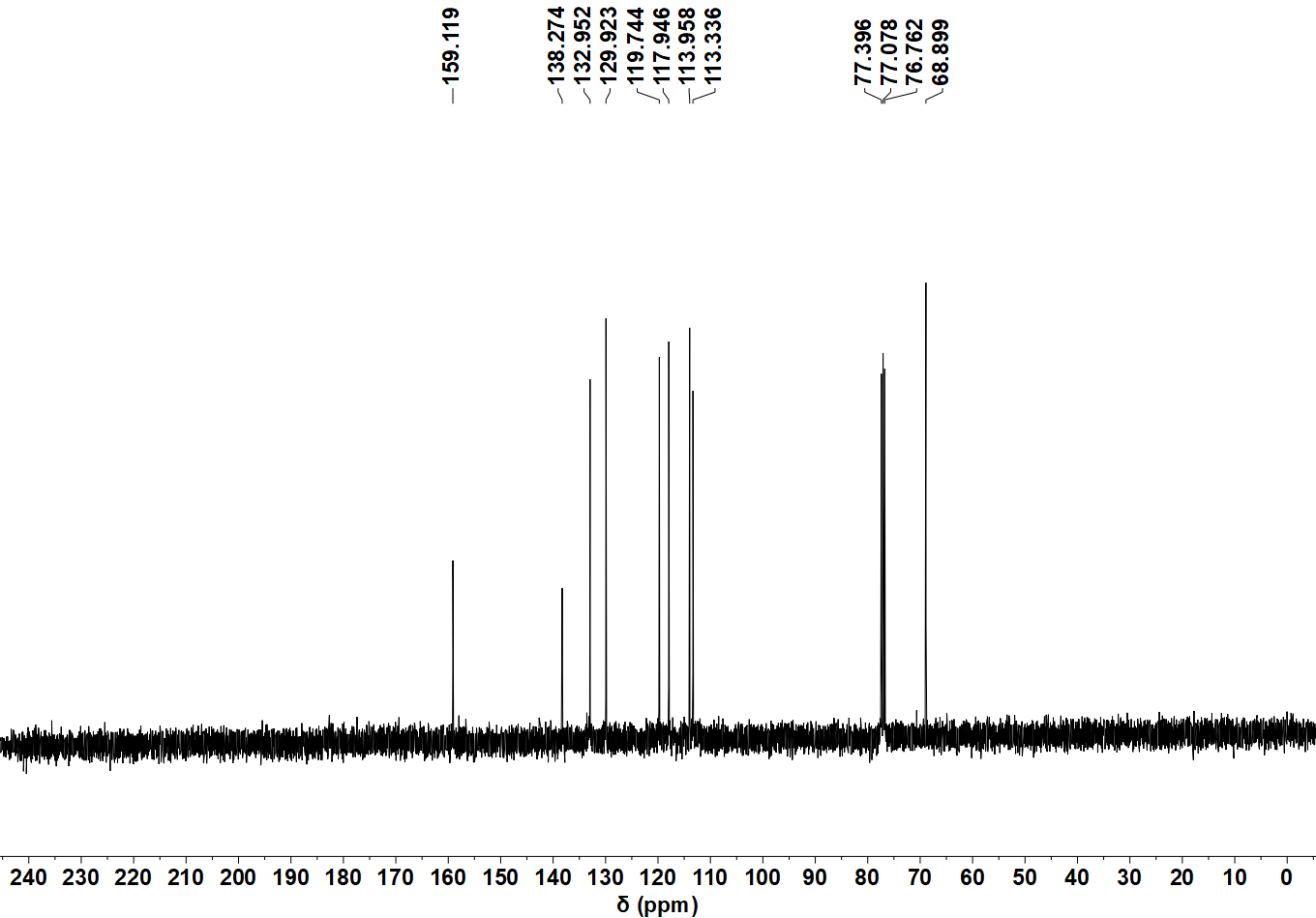


**Figure S2.** ^1^H and ^13^C NMR spectrum for **1b**.


**Figure S3. Monomer synthesis step 2**

*Procedure for Figure S3:* To a 250 mL oven-dried round-bottom flask equipped with a magnetic stir bar, compound **1b** (3.0 g, 9.1 mmol, 1.0 equiv) was dissolved in dichloromethane (DCM, 50 mL). In a separate vessel, Grubbs catalyst (second generation) (772.6 mg, 0.91 mmol, 10 mol%) was dissolved in DCM (30 mL) and the resulting solution was added dropwise to the flask at room temperature. The reaction mixture was then heated to reflux and stirred for 4 hours. Reaction progress was monitored by TLC. Upon completion, the solvent was removed under reduced pressure, and the crude product was purified by column chromatography to afford the desired product **SS**.

(*E*)-5,10-dioxa-2,3-dithia-1,4(1,3)-dibenzenacyclodecaphan-7-ene (**SS**)

The product was isolated by column chromatography (hexane/ethyl acetate = 40:1, Rf = 0.6) as a white solid in 67 % yield. melting point (m.p.) 160 °C – 162 °C.

^1^H NMR (400 MHz, CDCl_3_) δ 7.38 – 7.26 (m, 2H), 7.02 (dt, J = 7.6, 2.2 Hz, 1H), 6.90 (t, J = 2.0 Hz, 1H), 5.81 (t, J = 2.1 Hz, 1H), 4.68 (s, 2H).

^13^C NMR (101 MHz, CDCl_3_) δ 157.62, 138.09, 130.35, 128.72, 126.83, 120.17, 119.71, 67.88.

HRMS (ESI): Calcd for C_16_H_15_O_2_S_2_ [M+H]^+^: 303.0513; found: 303.0514.


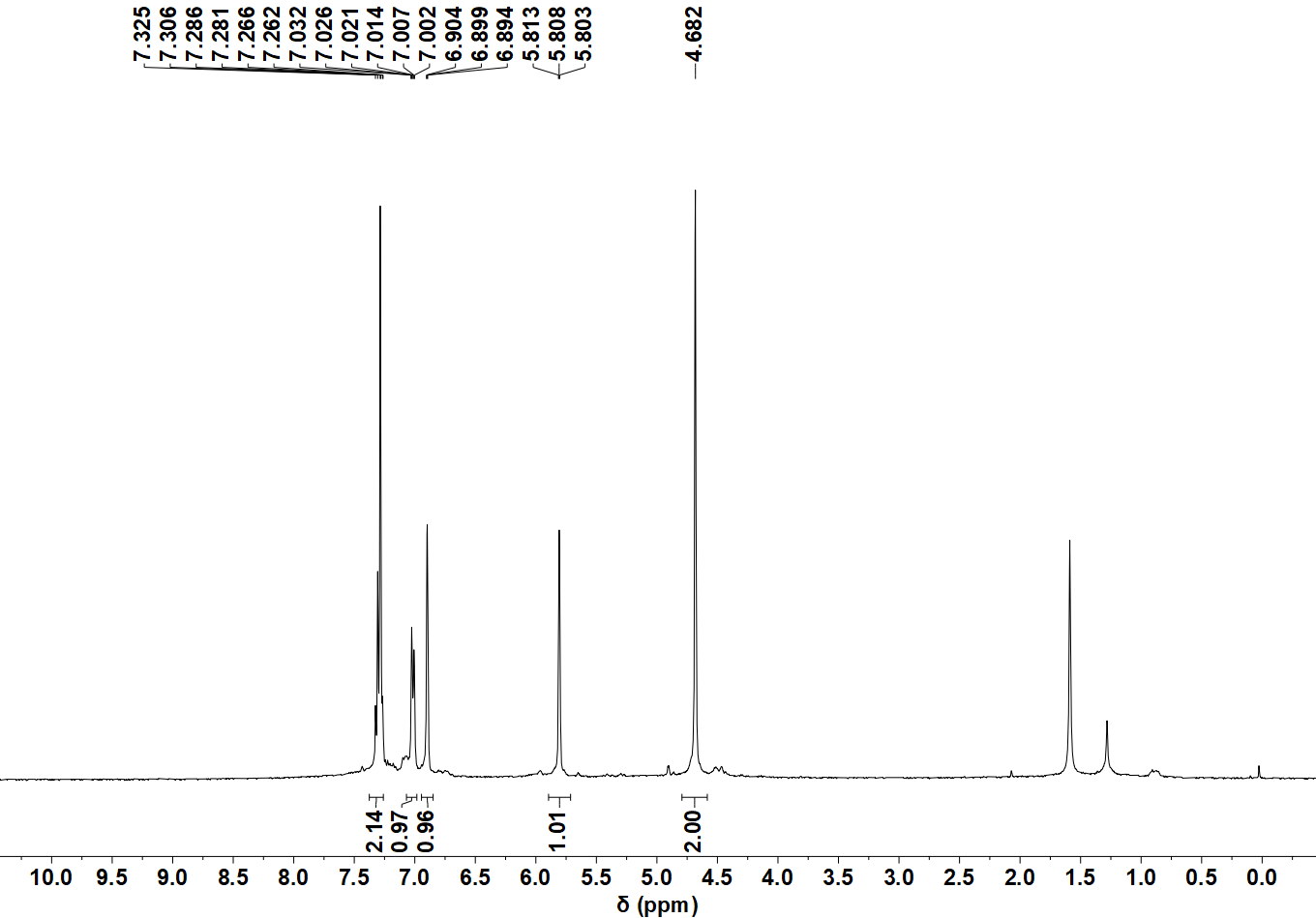


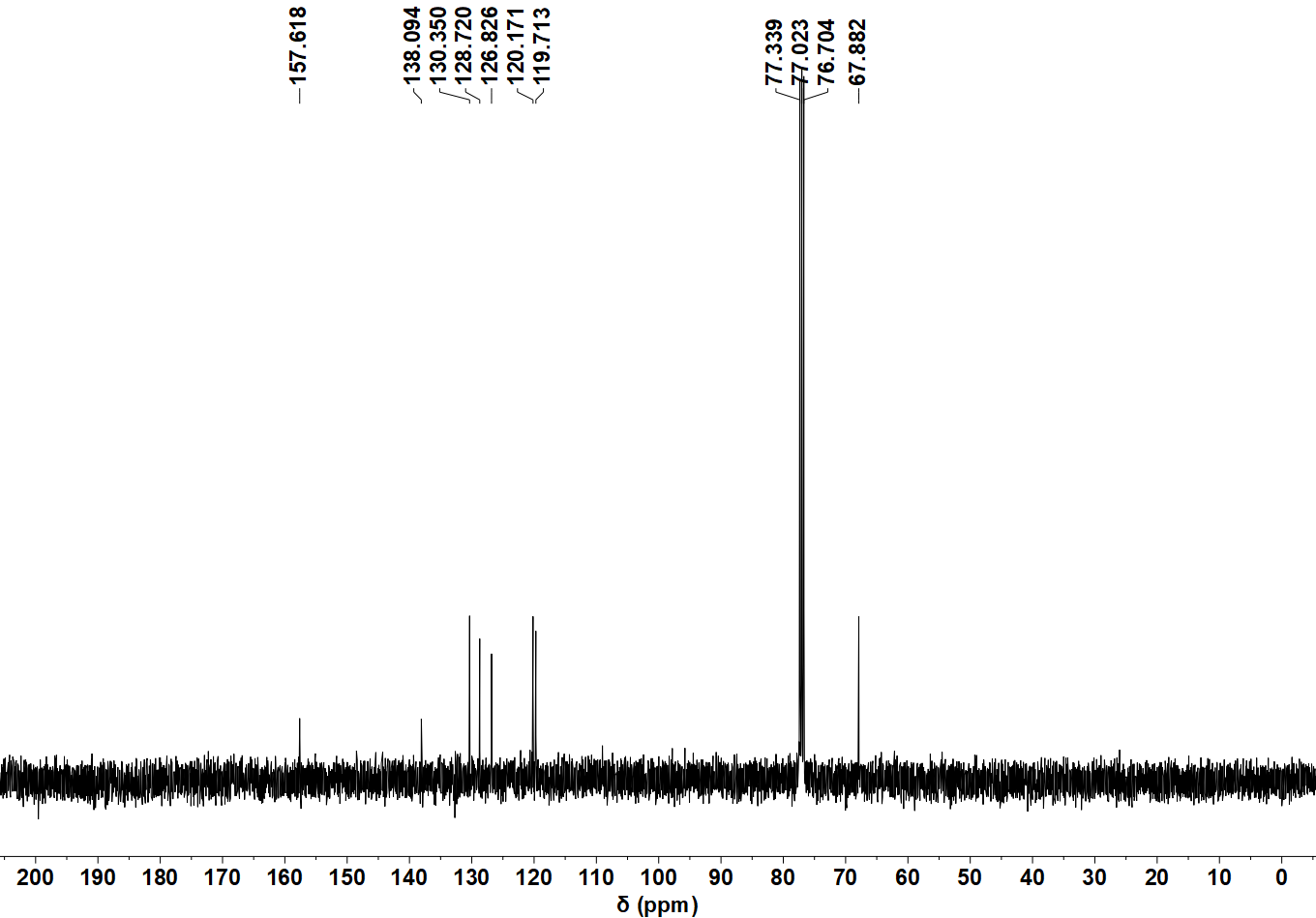


**Figure S4.** ^1^H and ^13^C NMR spectrum of monomer **SS**

**Crystal Structure Report for SS**

A colorless, plate-like specimen of C16H14O2S2, approximate dimensions 0.060 mm x 0.200 mm x 0.220 mm, was used for the X-ray crystallographic analysis. The X-ray intensity data were measured (λ = 0.71073 Å).


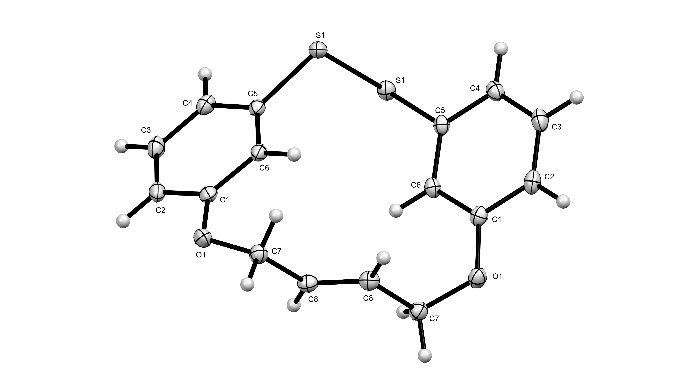


**Figure S5.** Crystal structures of monomer **SS**

**Table S1A: Data Collection Details for SS.**

| **Axis** | **dx/mm** | **2θ/°** | **ω/°** | **φ/°** | **χ/°** | **Width/°** |
| --- | --- | --- | --- | --- | --- | --- |
| Phi | 38.067 | 0.00 | 0.00 | 0.00 | 54.74 | 1.00 |
| Phi | 38.067 | 13.99 | -80.03 | 20.00 | 24.00 | 1.40 |
| Omega | 38.067 | 13.99 | 14.16 | 72.00 | -44.50 | 1.40 |
| Phi | 38.067 | 13.99 | 13.57 | 141.22 | -44.50 | 1.40 |

**Table S1B: Data Collection Details for SS.**

| **Frames** | **Time/s** | **Wavelength/Å** | **Voltage/kV** | **Current/mA** | **Temperature/K** |
| --- | --- | --- | --- | --- | --- |
| 180 | 1.00 | 0.71073 | 50 | 1.4 | 100 |
| 137 | 1.50 | 0.71073 | 50 | 1.4 | 100 |
| 74 | 1.50 | 0.71073 | 50 | 1.4 | 100 |
| 167 | 1.50 | 0.71073 | 50 | 1.4 | 100 |

**Table S2. Sample and crystal data for SS.**

| **Parameter** | **Value** |
| --- | --- |
| Identification code | ltp362m |
| Chemical formula | C₁₆H₁₄O₂S₂ |
| Formula weight | 302.39 g/mol |
| Temperature | 100(2) K |
| Wavelength | 0.71073 Å |
| Crystal size | 0.060 x 0.200 x 0.220 mm |
| Crystal habit | colorless plate |
| Crystal system | monoclinic |
| Space group | C 1 2/c 1 |
| Unit cell dimensions | a = 7.6830(8) Å α = 90° b = 15.0754(15) Å β = 91.879(4)° c = 11.7235(12) Å γ = 90° |
| Volume | 1357.1(2) Å³ |
| Z | 4 |
| Density (calculated) | 1.480 g/cm³ |
| Absorption coefficient | 0.390 mm⁻¹ |
| F(000) | 632 |

**Table S3. Atomic coordinates and equivalent isotropic atomic displacement parameters (Å²) for SS.**

U(eq) is defined as one third of the trace of the orthogonalized Uij tensor.

|  | **x/a** | **y/b** | **z/c** | **U(eq)** |
| --- | --- | --- | --- | --- |
| C1 | 0.45473(17) | 0.31844(9) | 0.49192(12) | 0.0156(3) |
| C2 | 0.54005(18) | 0.28823(10) | 0.59170(12) | 0.0186(3) |
| C3 | 0.58479(19) | 0.19960(11) | 0.60211(12) | 0.0205(3) |
| C4 | 0.54649(18) | 0.13964(10) | 0.51434(12) | 0.0177(3) |
| C5 | 0.46262(17) | 0.17059(9) | 0.41481(11) | 0.0145(3) |
| C6 | 0.41669(17) | 0.26008(9) | 0.40229(11) | 0.0151(3) |
| C7 | 0.36022(19) | 0.44760(9) | 0.38633(12) | 0.0174(3) |
| C8 | 0.50922(18) | 0.44635(9) | 0.30617(12) | 0.0174(3) |
| O1 | 0.40876(14) | 0.40635(7) | 0.49298(9) | 0.0187(2) |
| S1 | 0.39874(4) | 0.09337(2) | 0.30606(3) | 0.01625(13) |

**Table S4. Bond lengths (Å) for SS.**

| **Bond** | **Length (Å)** | **Bond** | **Length (Å)** |
| --- | --- | --- | --- |
| C1–O1 | 1.3716(17) | C1–C6 | 1.3942(19) |
| C1–C2 | 1.398(2) | C2–C3 | 1.384(2) |
| C3–C4 | 1.394(2) | C4–C5 | 1.3947(19) |
| C5–C6 | 1.4009(19) | C5–S1 | 1.7833(14) |
| C7–O1 | 1.4347(18) | C7–C8 | 1.505(2) |
| C8–C8#1 | 1.320(3) | S1–S1#1 | 2.0694(7) |

**Table S5. Bond angles (°) for SS.**

| **Angle** | **Value (°)** | **Angle** | **Value (°)** |
| --- | --- | --- | --- |
| O1–C1–C6 | 124.68(13) | O1–C1–C2 | 114.89(12) |
| C6–C1–C2 | 120.39(13) | C3–C2–C1 | 119.77(13) |
| C2–C3–C4 | 121.01(13) | C3–C4–C5 | 118.77(13) |
| C4–C5–C6 | 121.18(13) | C4–C5–S1 | 119.24(11) |
| C6–C5–S1 | 119.42(10) | C1–C6–C5 | 118.88(12) |
| O1–C7–C8 | 110.94(11) | C8#1–C8–C7 | 124.34(17) |
| C1–O1–C7 | 118.07(11) | C5–S1–S1#1 | 105.01(5) |

**Table S6. Hydrogen atomic coordinates and isotropic atomic displacement parameters (Å²) for SS.**

|  | **x/a** | **y/b** | **z/c** | **U(eq)** |
| --- | --- | --- | --- | --- |
| H2 | 0.5672 | 0.3284 | 0.6521 | 0.022000 |
| H3 | 0.6425 | 0.1793 | 0.6701 | 0.025000 |
| H4 | 0.5769 | 0.0788 | 0.5222 | 0.021000 |
| H6 | 0.3606 | 0.2806 | 0.3339 | 0.018000 |
| H7A | 0.3247 | 0.5097 | 0.4001 | 0.021000 |
| H7B | 0.2594 | 0.4159 | 0.3509 | 0.021000 |
| H8 | 0.6242 | 0.4455 | 0.3386 | 0.021000 |

**III. Synthesis of the polymers**

All pDCPD samples were prepared as thin films using the following general procedure. A designated amount of monomer **SS** (100 mg, 0, 0.025, 0.05, 0.1, or 0.2 equiv.) was mixed with DCPD (1.0 equiv.) and dissolved in 5 mL of dichloromethane (DCM) to afford a clear, colorless solution. To this solution, 0.5 mL of a stock solution of Grubbs catalyst (second generation, 4 mg/mL in DCM) was added, resulting in the formation of a homogeneous pink solution. The mixture was then transferred into a Ø 60 mm petri dish and allowed to stand at room temperature to facilitate slow evaporation of the solvent. This process yielded a uniform film ranging from colorless to pale yellow. The film was carefully removed from the dish using tweezers and subsequently placed in a vacuum oven at 160 °C for 1 hour to complete the curing process.

**
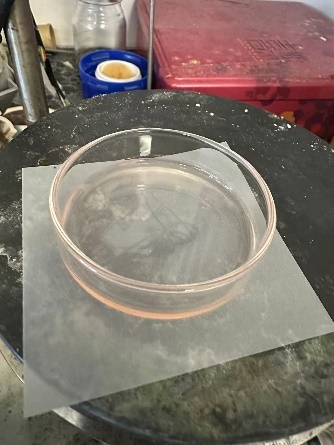

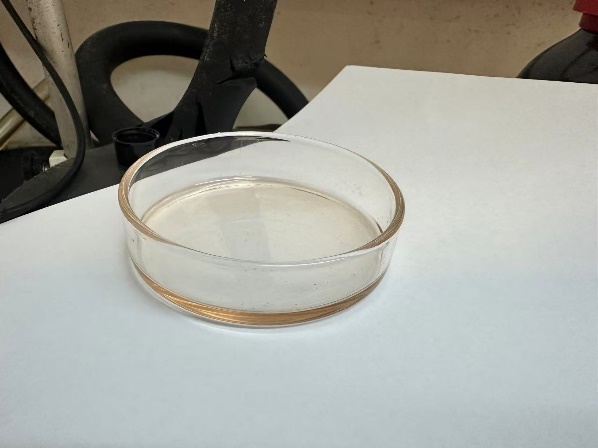

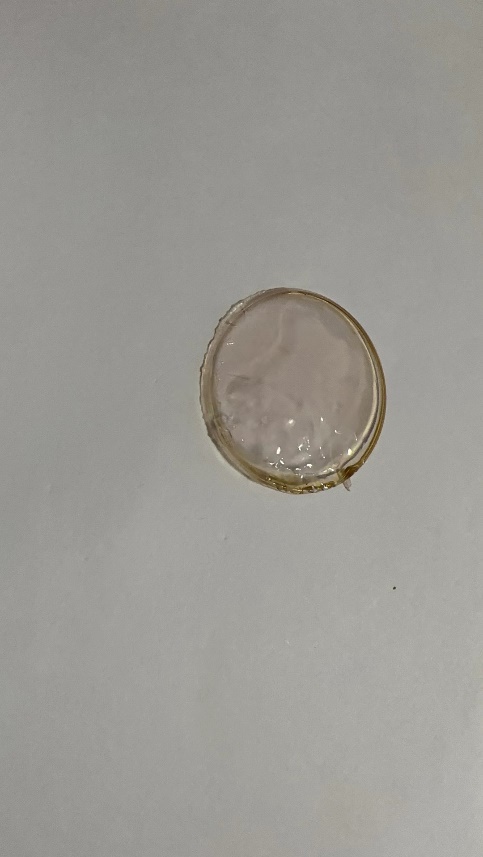

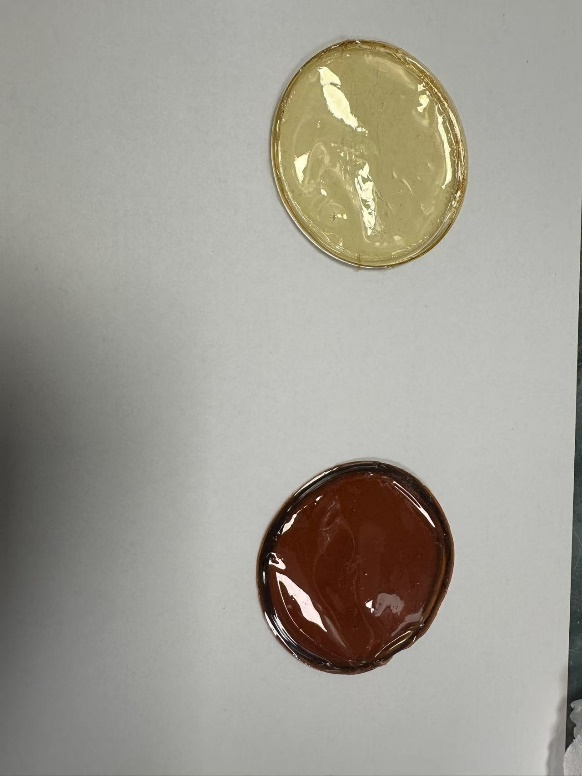
**

**Figure S6.** Representative photograph illustrating the polymerization procedure.

**Elemental analysis of SS-pD-5 sample**

Elemental analysis was performed to quantify the sulfur content in the SS-pD-5 sample and to further verify the incorporation of the sulfur-containing SS comonomer into the pDCPD network. The measurement was conducted using a PerkinElmer 2400 Series II CHNS/O Elemental Analyzer. The solid SS-pD-5 sample was dried under vacuum prior to analysis to remove residual solvent and moisture.

**Table S7. Elemental analysis result of the SS-pD-5 sample**

| **Sample** | **C / wt%** | **H / wt%** | **N / wt%** | **S / wt%** |
| --- | --- | --- | --- | --- |
| SS-pD-5 | 68.76 | 6.96 | 0.12 | 2.05 |

**XPS analysis of SS-pD-5 sample**

XPS analysis was performed to further verify the presence of sulfur in the SS-pD-5 network. The high-resolution S 2p spectrum of SS-pD-5 exhibited a distinct signal in the 163–165 eV region, which is characteristic of non-oxidized sulfur species such as C–S/S–S bonds. The appearance of this S 2p signal confirms the presence of sulfur in the SS-pD-5 sample and supports the successful incorporation of the disulfide-containing SS comonomer into the pDCPD network.

**Figure S7**. High-resolution S 2p XPS spectrum of **SS**-pD-5. The distinct signal in the 163–165 eV region is consistent with non-oxidized sulfur species such as C–S/S–S bonds, supporting the incorporation of the disulfide-containing SS comonomer into the pDCPD network.

**IV. Swelling experiments and gel content tests**

For each sample, approximately 100 mg of the cured material was placed into a 20 mL vial containing tetrahydrofuran (THF) and incubated at 50 °C for 48 hours to allow for solvent extraction of the soluble fraction. After incubation, the samples were washed with fresh THF three times to ensure complete removal of any extractable material. The remaining insoluble residue was then dried in a vacuum oven at 80 °C for 12 hours. The initial mass of the sample prior to extraction was recorded as m₀, and the final dry mass as m₁. The gel content was calculated using the following equation:

Gel content (%) = 100 × (m₁ / m₀)


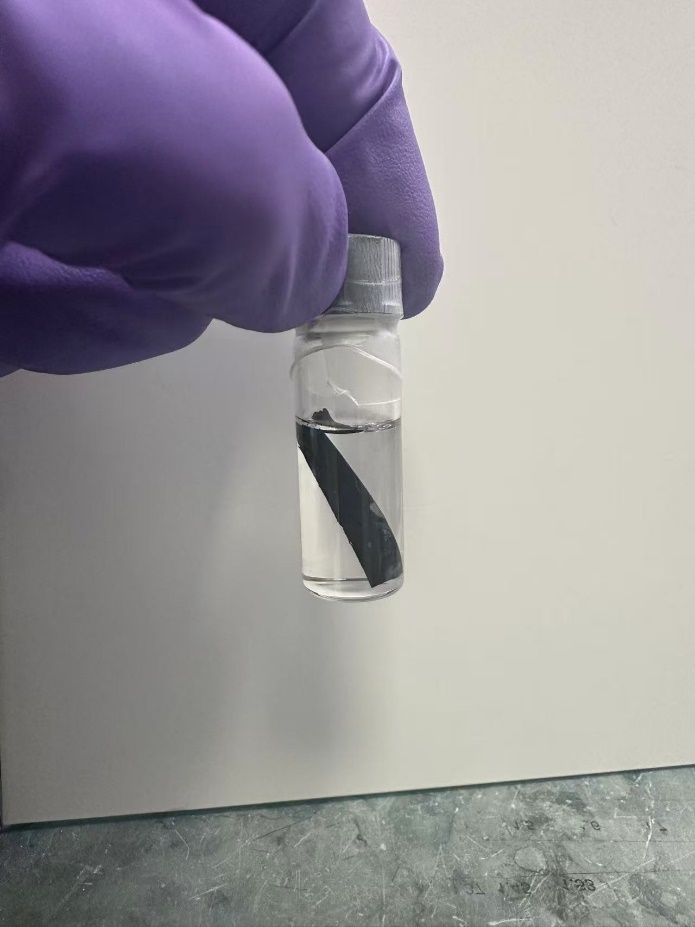

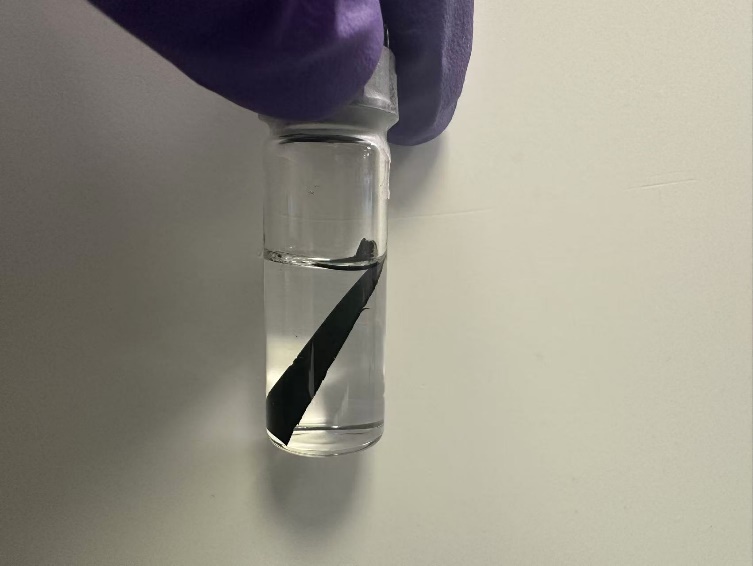


**Figure S8.** Representative photographs of **SS**-pD samples before (left) and after swelling in THF at 50 °C for 48 hours.

**V. Degradation tests and quantification of residue mass**

Samples of SS-doped pDCPD (100 mg each), prepared with varying monomer ratios, were cut into small pieces and placed into a 50 mL round-bottom flask containing 500 mg of dimethyldisulfide and 20 mL of tetrahydrofuran (THF). To this suspension, hydroxy(tosyloxy)iodobenzene (HTIB, 50 mg) was added in one portion. The reaction mixture was stirred at 70 °C for 24 hours, during which the solution gradually turned yellow and the polymer films degraded into fine powders. Upon completion, the reaction mixture was filtered. The filtrate was retained for further characterization and downstream experiments. The solid residue was washed with THF (3 × 10 mL) and the solids were dried in a vacuum oven at room temperature for 24 hours. The final mass of the dried solids was recorded and compared to the initial sample mass to evaluate the extent of degradation.

**VI. Functional performance characterization of poylmers**

**Dynamic mechanical analysis (DMA) tests**

Rectangular polymer samples with approximate dimensions of 20 mm (length) × 5 mm (width) × 0.15 mm (thickness) were equilibrated at 30 °C for 5 minutes, followed by heating at a rate of 3 °C min⁻¹ under an oscillation frequency of 1 Hz.

**Thermogravimetric analysis (TGA) tests.**

Approximately 10 mg of sample was heated from 30 to 800 °C at a rate of 20 °C/min under a nitrogen flow (60 mL/min).

**Tensile tests**

pDCPD samples were cut into rectangular strips with approximate dimensions of 30 mm (length) × 5 mm (width) × 0.15 mm (thickness). Tensile tests were performed at room temperature (RT) using a strain rate of 5% min⁻¹ until fracture. The tensile properties of each sample were determined by averaging the results from three independent measurements.

**Calculation of Apparent Crosslinking Density of Thermoset Networks**

The apparent crosslinking density of the thermoset networks was estimated from the rubbery storage modulus according to rubber elasticity theory using the following equation:

$$\rho=\frac{E^{'}}{3RT}$$

where $\rho$is the apparent crosslinking density, $E^{'}$is the storage modulus of the thermoset network measured at $T_{g}+{40}^{\circ}C$, $R$is the gas constant $\left( 8.314 J mol^{-1} K^{-1} \right)$, and $T$is the absolute temperature in Kelvin.

**Table S8. Calculation Details for Apparent Crosslinking Density**

| **Sample** | $T_{g}+{40}^{\circ}C$**/ °C** | $E^{'}$**at** $T_{g}+{40}^{\circ}C$**/ MPa** | **crosslinking density / mol m⁻³** |
| --- | --- | --- | --- |
| pDCPD | 219.4 | 211 | $1.72\times{10}^{4}$ |
| SS-pD-5 | 224.3 | 194 | $1.56\times{10}^{4}$ |
| SS-pD-20 | 196.4 | 99 | $8.45\times{10}^{3}$ |

**VII. Wide-Angle X-ray Scattering (WAXS) Characterization**

WAXS measurements were performed to investigate the local packing structures of pristine pDCPD and SS-doped pDCPD networks. The samples were measured in the solid state at room temperature using Cu Kα radiation. The scattering profiles were collected over the selected 2θ range and processed for comparison among different SS contents. Particular attention was paid to the 22–26° region, which corresponds to d-spacings of approximately 4.0–3.4 Å based on Bragg’s law. This region was used to evaluate possible short-range aromatic packing in the SS-containing networks.

**Figure S9**. WAXS spectra of pristine pDCPD and SS-doped pDCPD networks in the 22–26° region. The enhanced broad scattering of SS-containing samples suggests increased short-range aromatic packing.

**VIII. Malleability and reprocessability of SS-doped pDCPD samples**

5 mol% SS-doped pDCPD sample was chosen as the model polymer for recycling experiments.

**Stress relaxation measurements and activation energy calculation**

The rectangular sample was preloaded with a 1 × 10⁻³ N force to ensure alignment and equilibrated at the target temperature for 5 minutes. It was then subjected to a constant 2% strain using a dynamic mechanical analyzer (DMA). The strain was held throughout the test, and the decay of the relaxation modulus was recorded over time.

Activation energies (Eₐ) were determined using the methodology reported in literature^1,2,3^.

The relaxation time τ* refers to the time at which the relaxation modulus decreases to 1/e (~36.7%) at temperatures ranging from 160 to 190 °C. The activation energy (Eₐ) of the solid-state bond exchange was calculated via the Arrhenius equation (Eq. S1).

τ*(T) = τ₀ exp(Eₐ / RT) (Eq. S1)

y = 8.33x-14.05

E_a_= 69 kJ/mol

**Figure S10**. Fitting of the relaxation time to Arrhenius’ equation.

**Reprocessing process**

A 5 mol% **SS**-pDCPD film was cut into small pieces and placed between two steel plates lined with PTFE sheets. The assembly was then hot-pressed at 180 °C under a pressure of 30 bar for 10 minutes. Upon cooling to room temperature, a defect-free, brown-colored film was obtained. The same reprocessing procedure was repeated for the second and third cycle. All the reprocessed films were subjected to tensile testing and IR testing under the same conditions as the original sample.


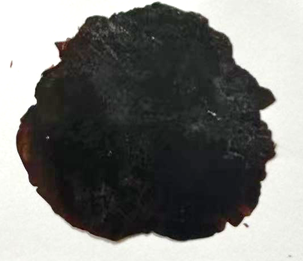

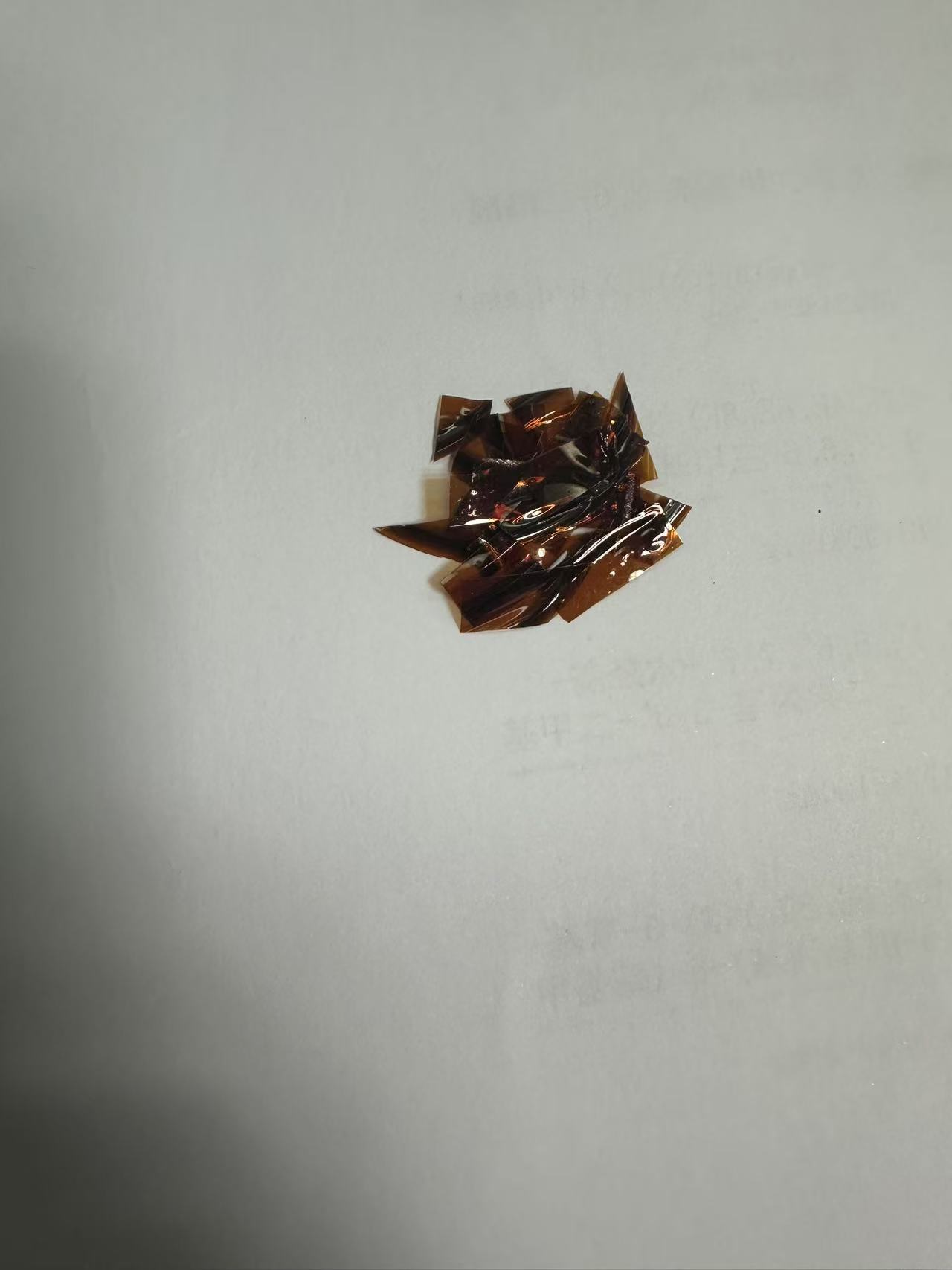

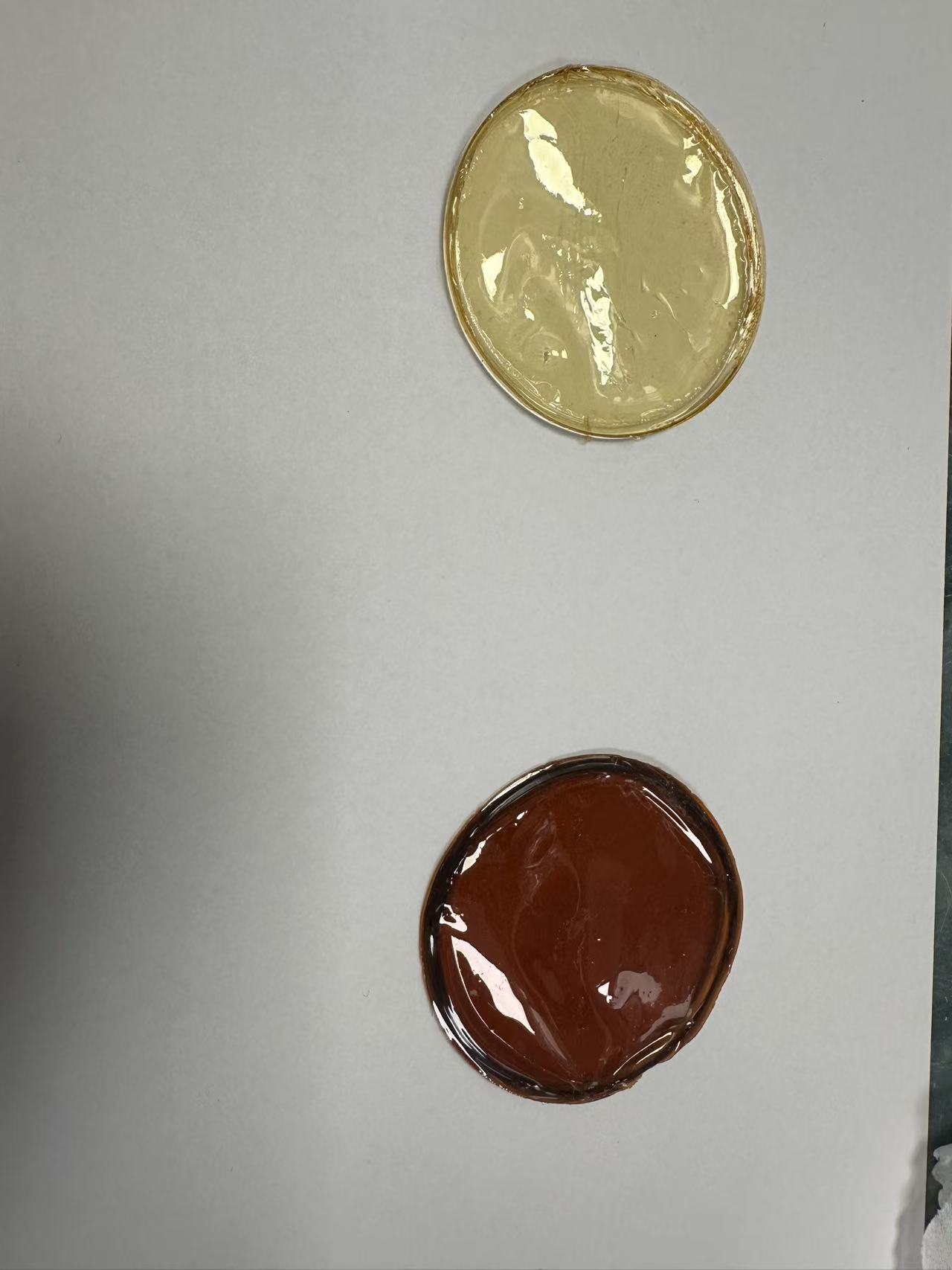


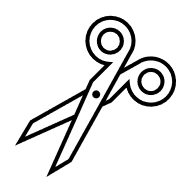


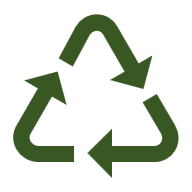


**Figure S11**. photographic illustration of the thermal reprocessing product for SS-pDCPD samples

**Figure S12**. Representative tensile stress–strain curves comparing the original and reprocessed samples.

**IX. Recovery of degraded SS-doped pDCPD fragments and characterization**

**Isolation of Dissolved SS-pDCPD fragements**

The solutions obtained from the degradation experiments were concentrated under reduced pressure. The resulting degradation fragments were redissolved in warm dichloromethane (DCM) and subsequently recrystallized to afford a white solid. The solid was collected by filtration and dried in a vacuum oven at room temperature for 24 hours.

**GPC analysis of SS-pDCPD fragements**

The fragments were dissolved in THF at a concentration of 1 mg/mL. The material was then filtered through a 0.2 µm Teflon filter before analysis. Gel permeation chromatography (GPC) analyses were performed on an Agilent 1260 Infinity system with columns using a THF mobile phase at a flow rate of 1 mL/min.

**Table S9: Calculated Fragment Molecular Weights from GPC-MALS**

| **Sample** | **M_w_** | **M_n_** | **M_w_/M_n_** |
| --- | --- | --- | --- |
| **SS-pD-2.5** | 6461 | 3920 | 1.64 |
| **SS-pD-5** | 2644 | 1937 | 1.36 |
| **SS-pD-10** | 2312 | 1726 | 1.33 |
| **SS-pD-20** | 1773 | 1298 | 1.36 |

**TEM Imaging of Degradation Fragments from 5 mol% SS-pDCPD**

Recovered fragments (1 mg) were redissolved in 1 mL of ethanol, and 10 μL of the solution was drop-cast onto a TEM grid. After complete solvent evaporation, the residual solids were imaged using a JEM-1400 Flash transmission electron microscope.

**NMR characterization.**
The chemically degraded oligomeric fragments were characterized by solution-state NMR spectroscopy to further identify the structural features of the degradation products. After chemical degradation, the THF solution was concentrated by solvent evaporation, and the obtained residues were purified by low-temperature precipitation/filtration and dried under vacuum. The purified fragments were then dissolved in CDCl_3_ for NMR analysis.


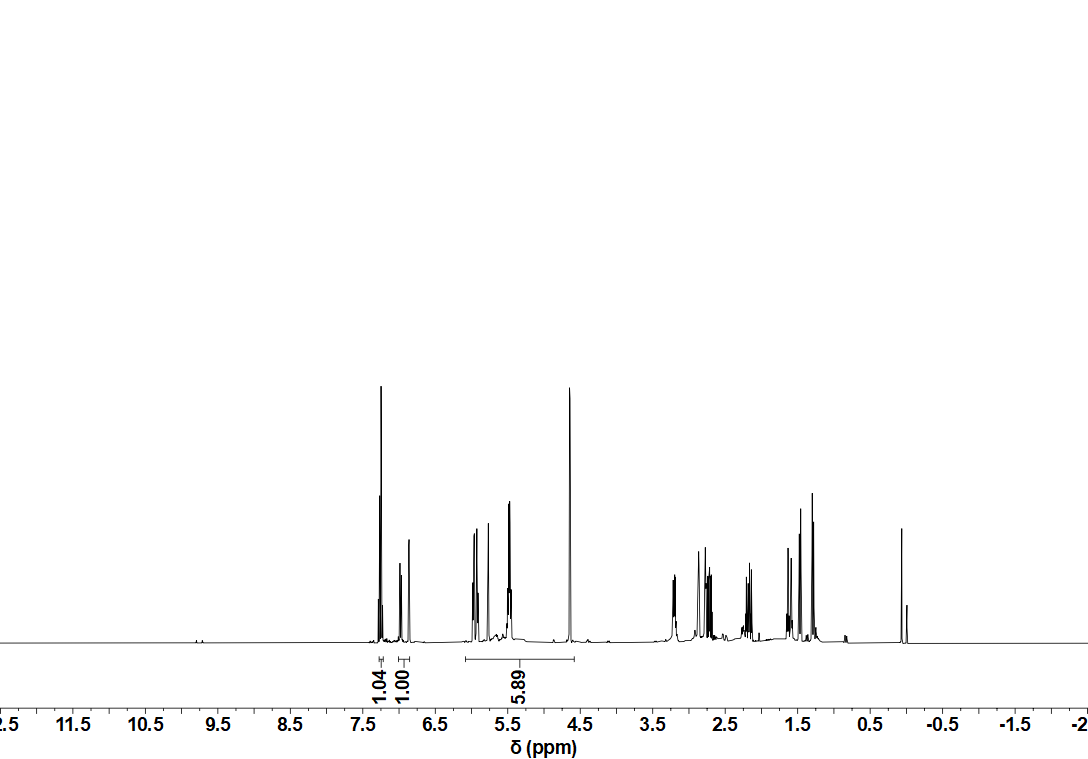


**Figure S13.** ¹H NMR spectrum of chemically degraded SS-pD-5 fragments, showing characteristic pDCPD-derived aliphatic/olefinic resonances and SS-derived aromatic resonances.

**DOSY NMR characterization.**

Diffusion-ordered spectroscopy (DOSY) NMR was performed to evaluate the solution-state diffusion behavior of the chemically degraded fragments and to distinguish polymer-derived oligomeric fragments from freely diffusing small-molecule residues. The DOSY experiment was conducted using a diffusion gradient duration δ = 2 ms and a diffusion time Δ = 100 ms.


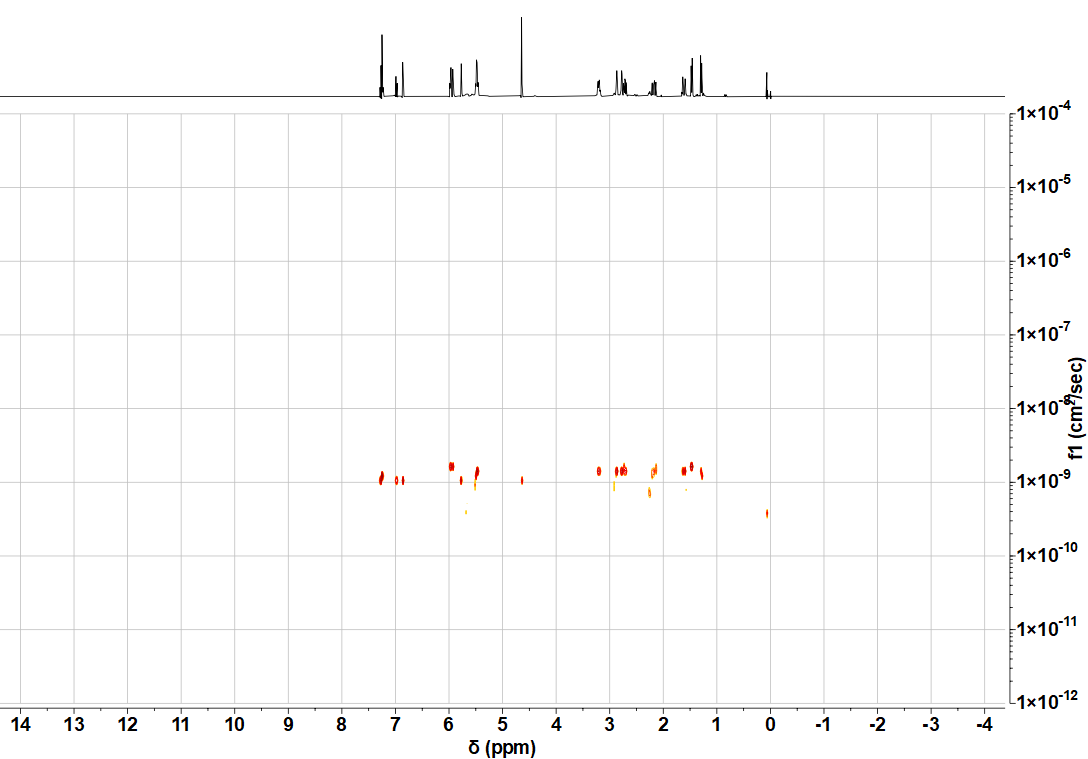


10^-9^ cm^2^s^-1^

**Figure S14.** DOSY NMR spectrum of chemically degraded **SS**-pD-5 fragments measured with δ = 2 ms and Δ = 100 ms. The comparable diffusion behavior of the major pDCPD-derived and SS-derived resonances supports their assignment to the same soluble oligomeric/polymeric fragment population.

**Dynamic light scattering (DLS) characterization.**

DLS measurements were performed to evaluate the solution-state size distribution of the chemically degraded products. The degraded oligomeric fragments were dissolved in toluene at a concentration of 2 mg mL⁻¹ and analyzed using a Malvern Nano-ZS Zetasizer at room temperature. Prior to measurement, the solution was gently mixed to ensure homogeneous dispersion of the soluble fragments.

The DLS results showed nanoscale species in solution with an average hydrodynamic diameter of approximately 20 nm, supporting the formation of soluble polymer-derived fragments after chemical degradation. This result provides complementary solution-state evidence to the GPC and TEM analyses, indicating that the crosslinked SS-doped pDCPD network was converted into dispersed oligomeric/polymeric fragments rather than remaining as an insoluble network residue.

**Figure S15.** DLS size distribution of chemically degraded SS-pD-5 fragments in toluene.

**X. Recycling of pDCPD fragments and characterization**

**Repolymerization of Degradation Fragments with DCPD**

Degraded fragments (200 mg) were combined with dicyclopentadiene (DCPD, 200 mg) and dissolved in 5 mL of dichloromethane (DCM) to form a clear, colorless solution. To this mixture, 0.5 mL of a stock solution of Grubbs’ second-generation catalyst (4 mg/mL in DCM) was added, producing a homogeneous pink solution. The reaction mixture was then transferred into a 60 mm diameter petri dish and left at room temperature to allow slow solvent evaporation, resulting in a uniform, colorless film. The dish was subsequently placed in a vacuum oven at 160 °C for 1 hour to complete the curing process. Upon cooling, the cured film was carefully removed using tweezers and subjected to tensile and IR spectroscopy analysis to assess its mechanical and chemical properties.

**
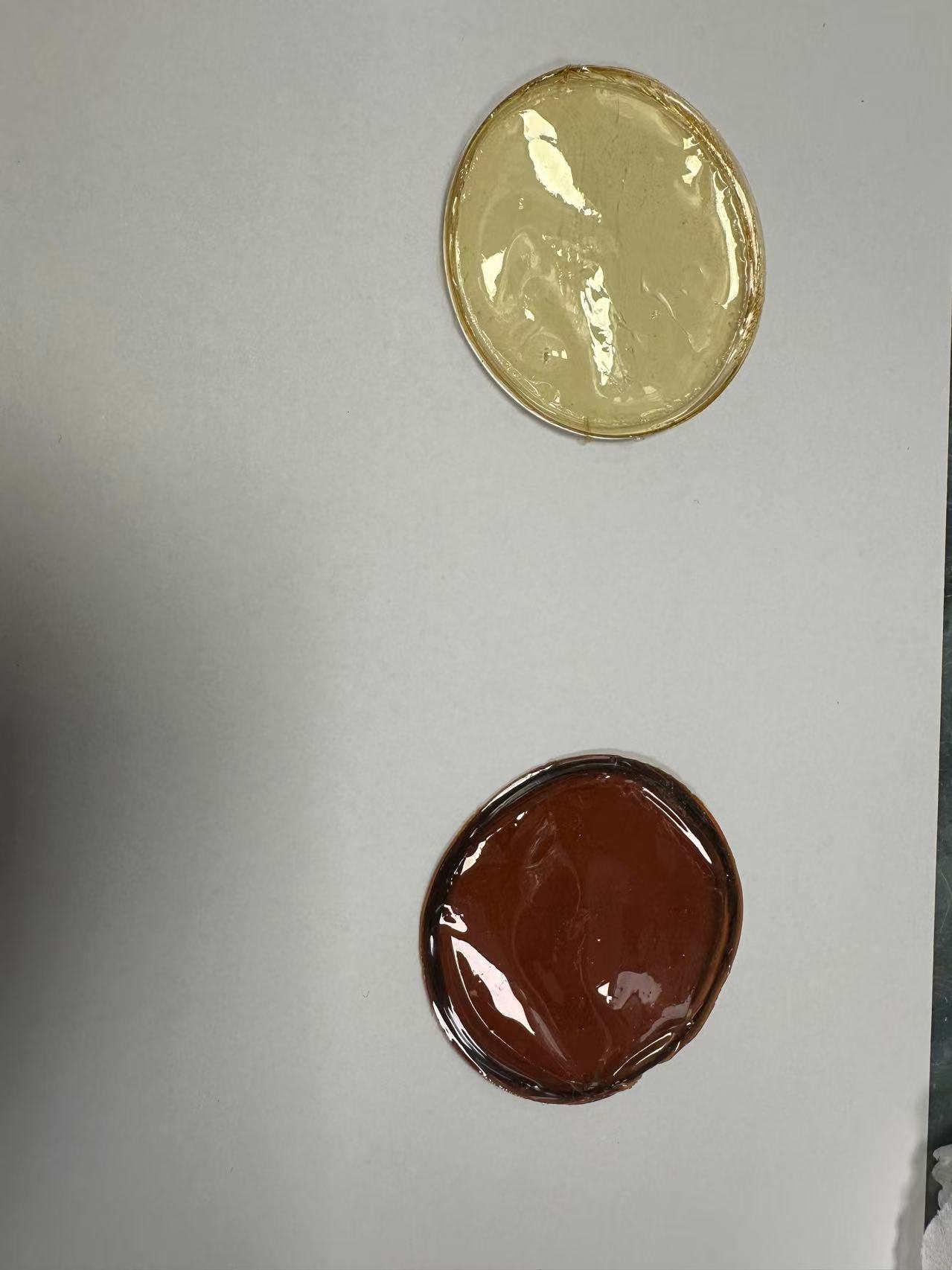
**

**Figure S16.** Photographs of recycled SS-pDCPD polymers. The color becomes progressively lighter due to the decreased content of the disulfide comonomer during recycling.

**
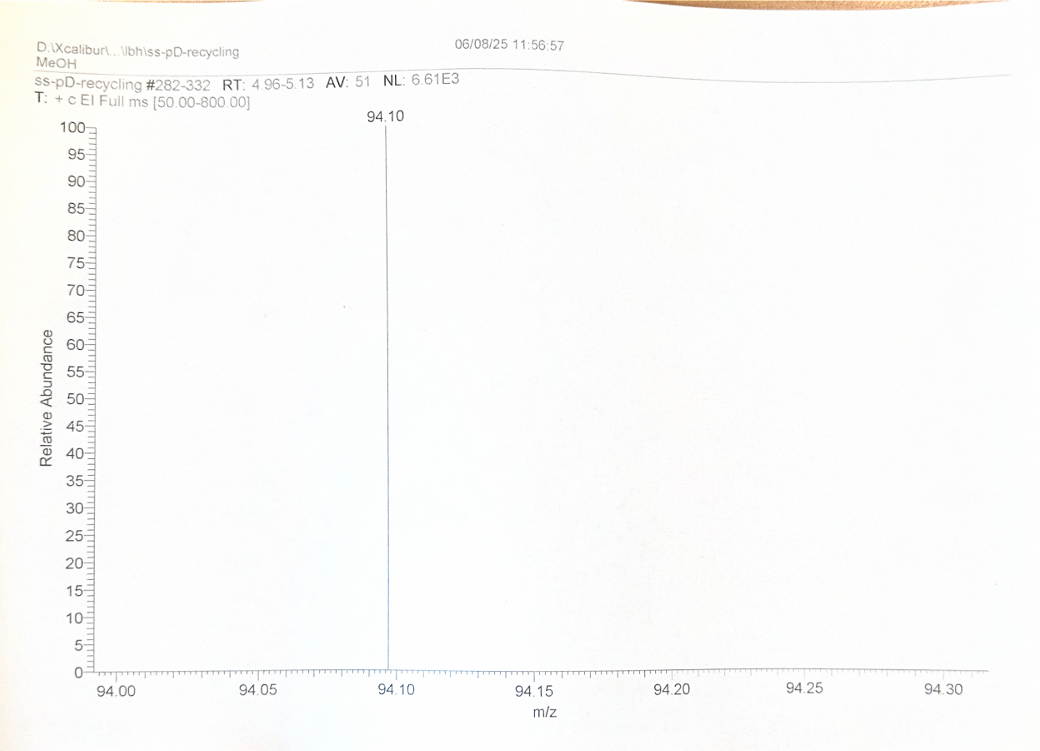
**

**Figure S17.** GC-MS data of dimethyl disulfide detected from the surface of the recycled polymer.

**Figure S18. Representative tensile stress–strain curves comparing the original and reprocessed samples.**

**XI. Comparison with State-of-the-Art pDCPD Recycling Systems**

**Table S10. Comparison of recyclability and key thermomechanical properties of representative pDCPD systems.**

| **Strategy** | **Young’s modulus (MPa)** | **Tg (°C)** | **Thermal reprocessability** | **Chemical recyclability** |
| --- | --- | --- | --- | --- |
| Conventional pDCPD | ~1000 | 180 | No | No selective chemical degradation |
| Silyl ether-doped pDCPD  (*Nature* 2020) | ~1000 ± 3 | 138 | No thermal reprocessing reported | TBAF-triggered degradation; 7.5–10 vol% iPrSi samples showed nearly complete mass loss; degradation products could be recycled into new pDCPD using 25 wt% fragments |
| SFC-containing pDCPD  (*ACIE* 2025) | N.A. | 163 ± 1 | No thermal reprocessing reported | TBAF-triggered deconstruction at much lower loading, as low as ~1 mol% SFC |
| Fragment-reactivated pDCPD  (*JACS* 2025) | r1: 1930 ± 93;  r2: 1780 ± 35;  r3: 1790 ± 73 | 130 ± 1 | No thermal reprocessing reported | Multi-generation chemical recycling with 40–45 wt% reactivated fragments per cycle |
| Disulfide-doped pDCPD (**SS**-pD-5 in This work) | 1732 | 182 | Yes; thermal reprocessing through dynamic disulfide exchange | Yes; chemical degradation via HTIB/DMDS in THF at 70 °C, followed by repolymerization/reconstruction |

**XII. References**

[1] Y. Nishimura, J. Chung, H. Muradyan, et al., “Silyl Ether as a Robust and Thermally Stable Dynamic Covalent Motif for Malleable Polymer Design,” *J. Am. Chem. Soc.* 139 (2017): 14881–14884.
https://doi.org/10.1021/jacs.7b08826

[2] J. Zheng, H. Feng, X. Zhang, et al., “Advancing Recyclable Thermosets through C═C/C═N Dynamic Covalent Metathesis Chemistry,” *J. Am. Chem. Soc.* 146 (2024): 21612–21622.
https://doi.org/10.1021/jacs.4c05346

[3] S. Wang, H. Feng, B. Li, et al., “Knoevenagel C═C Metathesis Enabled Glassy Vitrimers with High Rigidity, Toughness, and Malleability,” *J. Am. Chem. Soc.* 146 (2024): 16112–16118.
https://doi.org/10.1021/jacs.4c03503
